# Association between abdominal adiposity and cognitive decline in older adults: a 10-year community-based study

**DOI:** 10.1016/j.jnha.2024.100175

**Published:** 2024-02-02

**Authors:** Kazuaki Uchida, Taiki Sugimoto, Chikako Tange, Yukiko Nishita, Hiroshi Shimokata, Naoki Saji, Yujiro Kuroda, Nanae Matsumoto, Yoshinobu Kishino, Rei Ono, Toshihiro Akisue, Rei Otsuka, Takashi Sakurai

**Affiliations:** a Department of Prevention and Care Science, Research Institute, National Center for Geriatrics and Gerontology, Obu, Aichi 474-8511, Japan; b Department of Rehabilitation Science, Graduate School of Health Sciences, Kobe University, Kobe, Hyogo 654-0142, Japan; c Center for Comprehensive Care and Research on Memory Disorders, Hospital, National Center for Geriatrics and Gerontology, Obu, Aichi 474-8511, Japan; d Department of Epidemiology of Aging, Research Institute, National Center for Geriatrics and Gerontology, Obu, Aichi 474-8511, Japan; e Graduate School of Nutritional Sciences, Nagoya University of Arts and Sciences, Nisshin, Aichi 470-0196, Japan; f Department of Cognitive and Behavioral Science, Graduate School of Medicine, Nagoya University, Nagoya, Aichi 466-855, Japan; g Department of Physical Activity Research, National Institutes of Biomedical Innovation, Health and Nutrition, Settsu, Osaka 566-0002, Japan; h Department of Public Health, Graduate School of Health Sciences, Kobe University, Kobe, Hyogo 654-0142, Japan; i Research Institute, National Center for Geriatrics and Gerontology, Obu, Aichi 474-8511, Japan

**Keywords:** Abdominal obesity, Visceral fat, Subcutaneous fat, Cognitive decline, Older adults

## Abstract

**Objectives:**

This study aimed to investigate the association between abdominal adiposity and change in cognitive function in community-dwelling older adults.

**Design, Setting, and Participants:**

This longitudinal study included older adults aged ≥60 years without cognitive impairment who participated in the National Institute for Longevity Sciences – Longitudinal Study of Aging.

**Measurements:**

Cognitive function was evaluated biennially using the Mini-Mental State Examination (MMSE) over 10 years. Waist circumference (WC) was measured at the naval level, and subcutaneous fat area (SFA) and visceral fat area (VFA) were assessed using baseline computed tomography scans. WC, SFA, and VFA areas were stratified into sex-adjusted tertiles. A linear mixed model was applied separately for men and women.

**Results:**

This study included 873 older adults. In men, the groups with the highest levels of WC, SFA, and VFA exhibited a greater decline in MMSE score than the groups with the lowest levels (β [95% confidence interval]: WC, –0.12 [–0.23 to –0.01]; SFA, –0.13 [–0.24 to –0.02]; VFA, –0.11 [–0.22 to –0.01]). In women, the group with the highest level of WC and SFA showed a greater decline in MMSE score than the group with the lowest level (WC, –0.12 [–0.25 to –0.01]; SFA, –0.18 [–0.30 to –0.06]), but VFA was not associated with cognitive decline.

**Conclusion:**

Higher WC, SFA, and VFA in men and higher WC and SFA in women were identified as risk factors for cognitive decline in later life, suggesting that abdominal adiposity involved in cognitive decline may differ according to sex.

## 1 Introduction

The increasing rate of dementia is a rapidly growing global public health problem in aging society [1]. The number of individuals with dementia worldwide in 2019 was approximately 57.4 million, and it is estimated that this number will increase to 152.8 million by 2050 [2]. The global burden of dementia on health systems and societies is estimated to increase by fourfold between 2016 and 2060 [3]. Considering that the development of effective disease-modifying drugs is challenging, there is a growing need to identify modifiable risk factors for dementia [1].

Obesity in midlife is a modifiable risk factor for dementia [4]; however, in later life, a higher body mass index (BMI) has a protective effect [5]. This discordance in findings might be due to the reverse causation of weight loss beginning approximately 10 years before the onset of dementia [6,7]. It is also possible that BMI does not adequately reflect nutritional status (degree of obesity or weight loss) in later life [8]. This discrepancy arises because older adults usually experience changes in body composition in which fat mass increases and fat-free mass decreases with age, although the change in BMI is small [9]. In this context, recent studies have measured abdominal adiposity using waist circumference (WC) and waist-hip ratio, but not BMI, to assess nutritional status. Higher WC and waist-hip ratio are associated with the development of dementia and brain structural changes, such as hippocampal atrophy and white matter hyperintensity, in later life [10,11]. Based on the results of these studies, the accumulation of abdominal adiposity may have an adverse effect on brain health in later life.

Abdominal adiposity can be divided into two types: visceral and subcutaneous fat. Visceral fat accumulation affects brain structure through insulin resistance, inflammation, and vascular injury [12,13], which may be related to cognitive decline. To date, several observational studies have investigated the relationship between cognitive function and visceral and subcutaneous fat [[14], [15], [16], [17]], but the results are inconsistent and differ between the sexes according to the type of abdominal adiposity. Four studies have been reported regarding the association between visceral fat and cognitive function [[14], [15], [16], [17]]. One cross-sectional study reported in both sexes that a higher visceral fat area (VFA) was associated with lower cognitive function [14]. However, another cross-sectional study reported the opposite association in women: a higher visceral fat mass was associated with a lower rate of non-amnestic mild cognitive impairment (MCI) [15]. The other two studies reported no significant association in both sexes [16,17]. Three studies have been reported regarding the association between subcutaneous fat and cognition [14,16,17]. A longitudinal study reported that a higher subcutaneous fat area (SFA) was associated with greater cognitive decline in men [16]. One cross-sectional study reported in women that a higher SFA was associated with a decreased risk of dementia [17], whereas another study reported no significant association in both sexes [14]. Thus, there is no unified view on visceral and subcutaneous fat relationships with the cognitive function of each sex. In addition, while many studies from Caucasian populations have reported the relationship between abdominal adiposity and cognitive function, to our knowledge, only one study has examined the long-term impacts of visceral and subcutaneous fat on cognitive function in these studies [16]. Furthermore, as abdominal fat distribution differs between Caucasian and Japanese populations [18,19], it is necessary to accumulate the findings in Asia, including Japan.

This study investigated the association between abdominal adiposity at baseline and change in cognitive function in community-dwelling older adults using longitudinal data collected separately for men and women over 10 years.

## 2 Materials and methods

### 2.1 Data and study populations

This study was based on data collected as part of the National Institute for Longevity Sciences – Longitudinal Study of Aging (NILS-LSA), a community-based study involving community-dwelling older individuals [20]. NILS-LSA participants were randomly sampled according to age and sex from a population in Obu City and Higashiura Town in Aichi Prefecture, Japan. Participants were followed up every 2 years from the first wave (between November 1997 and April 2000) to the seventh wave (between July 2010 and July 2012). The baseline for this study was the second wave (April 2000 to May 2002) because Mini-Mental State Examination (MMSE) data were available starting from the second wave in the NILS-LSA.

Participants aged ≥60 years at the second wave (between April 2000 and May 2002) were included in this study. The exclusion criteria were older individuals who had cognitive impairment (MMSE < 24) in the second wave [21] (to reduce the impact of reverse causation), those who never participated in any of the third to seventh waves, and those with missing data for covariates at baseline.

This study was conducted according to the Declaration of Helsinki. This study was approved by the Ethics Committee of Human Research at the National Center for Geriatrics and Gerontology, Japan (No. 1525), and informed consent was obtained from all participants before study participation.

### 2.2 Measurements

#### 2.2.1 Cognitive function

Cognitive function was measured using the MMSE [22], administered to the participants during the second to seventh waves. Trained psychologists and graduate psychology students measured cognitive function. The MMSE has a scoring range of 0–30, with higher scores indicating better cognitive function.

#### 2.2.2 Abdominal adiposity

WC, SFA, and VFA in the second wave were used as abdominal adiposity indices. WC was measured at the naval level at the end of exhalation while the participant was in a standing position and not wearing a shirt. SFA and VFA at the L4–L5 level were measured using computed tomography (CT) (SCT-6800TX; Shimadzu, Japan). All CT scans were performed with participants in the supine position. SFA and VFA were calculated using computer software (Fat Scan; N2 Systems, Japan), as previously described [23]. In this study, WC, SFA, and VFA were stratified into sex-adjusted tertiles (lowest, middle, and highest groups), as described previously in a study that examined the association between abdominal adiposity and change in cognitive function [16].

#### 2.2.3 Other variables in the second wave

The following characteristics were recorded: age, sex, height, weight, fat-free mass, years of education, apolipoprotein E (APOE) genotype, current smoking status, and self-reported comorbidities (stroke, hypertension, dyslipidemia, diabetes mellitus, and cardiovascular disease). After measuring participants’ height (m) and weight (kg) using a digital scale, the Body Mass Index (BMI) was calculated as weight divided by height squared. Fat-free mass was measured using a QDR-4500 dual-energy X-ray absorptiometry (DXA) system (Hologic, Bedford, MA, USA), and the fat-free mass index (FFMI) was calculated as fat-free mass (kg) divided by height (m) squared. APOE genotypes were determined using polymerase chain reaction amplification [20], and participants were categorized as APOE-ε4 carriers and noncarriers.

### 2.3 Statistical analyses

To describe the characteristics of participants in the second wave, we calculated means ± standard deviation (SD) of age, years of education, MMSE, BMI, FFMI, WC, SFA, VFA, height, weight, and the total number of individuals (%) for other variables. We used the two-sample t-test for continuous variables and the chi-square test for categorical variables to examine sex differences. Moreover, we performed a t-test and chi-square test to compare the characteristics in the second wave between the older adults included in the main analysis (analyzed samples) and those who met exclusion criteria (excluded samples) in men and women. Furthermore, the means ± SD of each abdominal adiposity index were calculated in the groups with the lowest, middle, and highest levels of abdominal adiposity.

A linear mixed model allows for considering random participant-specific intercepts and the variability of MMSE scores during follow-up. In this analysis, the repeated MMSE score at each wave from baseline was used as the dependent variable, and the abdominal adiposity index at the second wave (ref. lowest group), time, and the interaction of abdominal adiposity indices and time were used as the independent variables. Time is expressed as waves from the second to the seventh waves. We performed the unadjusted model, the model that only age at second wave was adjusted (age-adjusted model), and the model that the following variables at the second wave were adjusted for confounding factors: age, the interaction of age and time, years of education, APOE-ε4, the interaction of APOE-ε4 and time, FFMI, current smoking status, and stroke, hypertension, dyslipidemia, diabetes mellitus, and cardiovascular disease (Full model). We calculated the fixed effects of independent variables with a random effect of intercept and slope. We calculated Pearson’s correlation coefficients between WC with SFA and VFA in this analysis. WC was not included in the model with SFA and VFA as independent variables because a strong correlation was found (Supplemental Tables S1 and S2).

In addition, to investigate the dose–response relationship between abdominal adiposity and cognitive decline, we calculated p for the trend across each abdominal adiposity index using the same statistical linear mixed model. The predicted change in the MMSE score over 10 years was calculated for the abdominal adiposity index groups, adjusted for confounding factors. Confounding factors were selected based on previous studies [[14], [15], [16], [17]]. APOE-ε4 was included as a confounding factor because it was reported to be a risk factor for cognitive and functional decline [24].

All analyses were performed separately for men and women because previous studies have reported a sex-specific association between abdominal adiposity and cognitive function [[14], [15], [16], [17]]. Statistical significance was set at p < 0.05; all analyses were performed using R version 3.6.1, and linear mixed models were performed using the R package -lme4 (https://cran.r-project.org/web/packages/lme4/index.html).

## 3 Results

### 3.1 Characteristics of the participants in the second wave

**Fig. 1:**
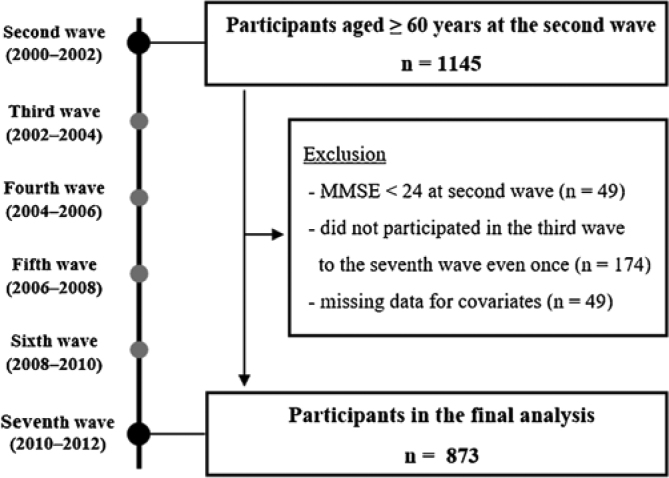
Flowchart of participant selection. Abbreviation: MMSE, Mini-Mental State Examination.

Fig. 1 shows a flowchart of participant selection for this study. In the second wave of the NILS-LSA, 1145 older adults aged ≥ 60 years were included. Older adults who met the following criteria were excluded: those with an MMSE score <24 in the second wave (n = 49), those who had never participated in the third to seventh waves (n = 174), and those with missing data for covariates (n = 49). Ultimately, 873 older adults were included in the analysis.

Table 1presents the baseline characteristics of the participants. The mean age of the 873 participants was 68.76 ± 5.65 years, and 52.5% of participants were men. The median follow-up period was 9.67 years. Among the participants, 87 (19.0%) men and 69 (16.6%) women were APOE-ε4 carriers. Men had a lower SFA (108.02 ± 43.89 cm² vs. 167.10 ± 64.83 cm²) and a higher VFA (97.11 ± 54.73 cm² vs. 73.69 ± 40.02 cm²) compared with women. Moreover, men had a lower prevalence of dyslipidemia and a higher prevalence of current smoking, stroke, and diabetes mellitus compared with women. The cumulative incidence of cognitive impairment (MMSE < 24 points) in the 10-year follow-up is 29 (6.3%) in men and 27 (6.5%) in women.

**Table 1:** Characteristics of the participants at baseline.

	All participants n = 873	Men n = 458	Women n = 415	*P* value
Age, year	68.76 ± 5.65	68.76 ± 5.76	68.77 ± 5.54	0.93
Years of education, year	10.88 ± 2.60	11.43 ± 2.80	10.27 ± 2.22	<0.01
APOE-ε4 carriers	156 (17.87)	87 (19.00)	69 (16.63)	0.41
MMSE, score	28.13 ± 1.60	28.07 ± 1.60	28.19 ± 1.59	0.21
BMI, kg/m^2^	22.83 ± 2.89	22.84 ± 2.75	22.82 ± 3.05	0.36
FFMI, kg/m^2^	16.93 ± 1.90	17.97 ± 1.66	15.79 ± 1.45	<0.01
Waist circumference, cm	84.89 ± 8.81	85.03 ± 8.07	84.67 ± 9.47	0.39
Subcutaneous fat area, cm^2^	136.10 ± 62.26	108.02 ± 43.89	167.10 ± 64.83	<0.01
Visceral fat area, cm^2^	85.98 ± 49.67	97.11 ± 54.73	73.69 ± 40.02	<0.01
Height, cm	156.42 ± 8.64	162.66 ± 5.71	149.54 ± 5.52	<0.01
Weight, kg	56.04 ± 9.39	60.51 ± 8.49	51.10 ± 7.72	<0.01
Current smoker	143 (16.38)	127 (27.73)	16 (3.86)	<0.01
Stroke	44 (5.04)	33 (7.21)	11 (2.65)	<0.01
Hypertension	319 (36.54)	164 (35.81)	155 (37.35)	0.69
Dyslipidemia	180 (20.62)	62 (13.54)	118 (28.43)	<0.01
Diabetes mellitus	80 (9.16)	53 (11.57)	27 (6.51)	0.01
Cardiovascular disease	133 (15.23)	78 (17.03)	55 (13.25)	0.15

Data are described as n (%) or mean ± standard deviation.

Abbreviations: APOE-ε4, apolipoprotein E-ε4; MMSE, Mini-Mental State Examination; BMI, body mass index; FFMI, fat-free mass index.

Supplementary Table S3 shows the comparison results of the characteristics in the second wave between the analyzed and excluded samples. In men, the excluded samples were older, had lower years of education, MMSE score, and height, and had a higher prevalence of stroke and diabetes mellitus than the analyzed samples. A similar trend was observed among women: the excluded samples were older, displayed lower MMSE scores and height, and had a higher prevalence of hypertension and cardiovascular disease than the analyzed samples.

### 3.2 The mean of abdominal adiposity in the lowest, middle, and highest groups

The mean abdominal adiposities for each sex in the groups with the lowest, middle, and highest adiposity levels are presented in Table 2. For the group with the highest abdominal adiposity, WC, SFA, and VFA in men were 93.61 ± 3.53 cm, 155.99 ± 27.55 cm^2^, and 159.54 ± 37.10 cm^2^, respectively. Those in women were 95.05 ± 4.85 cm, 239.52 ± 40.75 cm^2^, and 119.70 ± 26.89 cm^2^, respectively.

**Table 2:** Mean of abdominal adiposity in the lowest, middle, and highest groups.

		Abdominal adiposity index		
	Sex	Lowest group	Middle group	Highest group
Waist circumference, cm	Men	76.11 ± 5.07	85.42 ± 2.03	93.61 ± 3.53
	Women	74.38 ± 4.97	84.58 ± 2.63	95.05 ± 4.85
Subcutaneous fat area, cm^2^	Men	61.87 ± 21.83	106.51 ± 10.67	155.99 ± 27.55
	Women	99.12 ± 28.19	163.15 ± 15.88	239.52 ± 40.75
Visceral fat area, cm^2^	Men	40.73 ± 17.53	91.46 ± 14.18	159.54 ± 37.10
	Women	32.54 ± 10.84	69.14 ± 11.28	119.70 ± 26.89

Data are described as mean ± standard deviation.

### 3.3 Association between abdominal adiposity and change in cognitive function

Table 3presents the results of the multivariate linear mixed model with the full model, and Supplementary Tables 4 and 5 present the unadjusted and the age-adjusted model. The full model results were similar to those of the unadjusted and age-adjusted models. In men with the full model, the group with the highest level of abdominal adiposity showed a greater decline in MMSE score than the group with the lowest level (WC, β = –0.12, 95% confidence interval [CI] = –0.23 to –0.01; SFA, β = –0.13, 95% CI = –0.24 to –0.02; VFA, β = –0.11, 95% CI = –0.22 to –0.01). In women with the full model, those in the group with the highest WC and SFA showed a greater decline in MMSE score (WC, β = –0.12, 95% CI = –0.25 to –0.01; SFA, β = –0.18, 95% CI = –0.30 to –0.06) than those in the group with the lowest WC and SFA. While the trends for the association of WC and SFA with cognition were significant in both sexes (p < 0.05), VFA was only significant in men (p < 0.05).

**Table 3:** Association between abdominal adiposity and change in cognitive function: a linear mixed model with the full model.

	Men				Women			
	β	95% CI	*P* value	P for trend	β	95% CI	*P* value	P for trend
Waist circumference × time				0.01				0.04
Low	ref.				ref.			
Middle	0.03	–0.07 to 0.14	0.53		–0.07	–0.19 to 0.05	0.27	
High	–0.12	–0.23 to -0.01	0.03		–0.12	–0.25 to –0.01	0.05	
Subcutaneous fat area × time				0.02				<0.01
Low	ref.				ref.			
Middle	–0.04	–0.15 to 0.07	0.44		–0.11	–0.23 to 0.01	0.07	
High	–0.13	–0.24 to –0.02	0.02		–0.18	–0.30 to –0.06	<0.01	
Visceral fat area × time				0.04				0.59
Low	ref.				ref.			
Middle	0.02	–0.09 to 0.13	0.68		–0.08	–0.20 to 0.04	0.18	
High	–0.11	–0.22 to –0.01	0.04		0.03	–0.09 to 0.15	0.59	

Adjusted for age, the interaction of age and time, years of education, APOE-ε4, the interaction of APOE-ε4 and time, FFMI, current smoking status, stroke, hypertension, dyslipidemia, diabetes mellitus, and cardiovascular disease.

Abbreviations: FFMI, fat-free mass index; APOE-ε4, apolipoprotein E-ε4.

Note: In this linear mixed model, the repeated MMSE score at each wave from baseline was used as the dependent variable, and the abdominal adiposity index at the second wave (ref. lowest group), time, and the interaction of abdominal adiposity indices and time were used as the independent variables, and participant-specific intercepts and the variability of MMSE score during follow-up were considered as a random effect. Table 3 indicates only the results of the interaction between abdominal adiposity index and time.

**Fig. 2:**
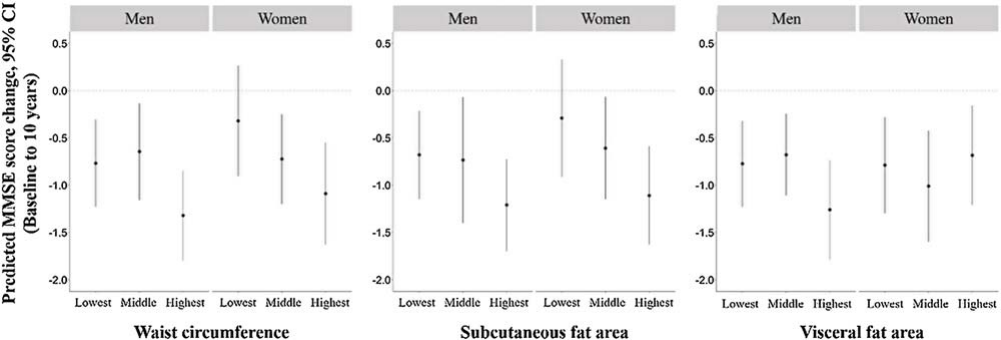
Predicted MMSE score change over 10 years. Adjusted for age, the interaction of age and time, years of education, APOE-ε4, the interaction of APOE-ε4 and time, FFMI, current smoking status, stroke, hypertension, dyslipidemia, diabetes mellitus, and cardiovascular disease Abbreviations: MMSE, Mini-Mental State Examination; FFMI, fat-free mass index; APOE-ε4, apolipoprotein E-ε4

Fig. 2 shows the predicted change in MMSE scores over 10 years for the groups with the lowest, middle, and highest levels of abdominal adiposity, adjusted for age, the interaction of age and time, years of education, APOE-ε4, the interaction of APOE-ε4 and time, FFMI, current smoking status, stroke, hypertension, dyslipidemia, diabetes mellitus, and cardiovascular disease. In men, the group with the highest WC, SFA, and VFA showed a larger decline in MMSE score than the group with the lowest WC, SFA, and VFA. In women, the group with the highest WC and SFA showed a larger decline in MMSE score than the group with the lowest WC and SFA.

## 4 Discussion

This study investigated the association between abdominal adiposity and change in cognitive function based on data from cognitively well-functioning community-dwelling older adults collected over 10 years. The results showed that even after adjusting for FFMI, higher WC, SFA, and VFA were associated with a more rapid decline in MMSE scores in men and higher WC and SFA were associated with a decline in MMSE scores in women.

To the best of our knowledge, only one longitudinal Health, Aging, and Body Composition study (Health ABC study) has examined the association between abdominal adiposity and change in cognitive function [16]. This previous study reported that a higher SFA, but not VFA, was associated with greater cognitive decline in men and that abdominal adiposity was not associated with cognitive decline in women [16]. This study is the first to show that higher VFA in men and higher SFA in women are associated with greater cognitive decline and that the type of abdominal fat associated with cognitive decline differs between men and women. Notably, these findings are based on Japanese individuals with smaller body sizes and fat masses than Westerners. In the previous study, the means ± SDs of WC in the groups with the lowest, middle, and highest levels of abdominal adiposity were 89.0 ± 8.0 cm, 100.4 ± 2.5 cm, and 112.9 ± 6.9 cm for men and 83.6 ± 6.6 cm, 97.8 ± 3.1 cm, and 113.2 ± 9.0 cm for women [16], respectively, which are higher than those of the participants in the current study. Our findings indicate the importance of abdominal adiposity, even in older Japanese adults with relatively lower abdominal fat.

One of the potential mechanisms underlying the association between visceral and subcutaneous fat and cognitive decline may be the abnormal secretion of adipokines. The adipose tissue is an endocrine organ that secretes adipokines that may be involved in vascular injury, atherosclerosis, and insulin resistance [25]. Among adipokines, inflammatory cytokines, such as interleukin-6 (IL-6) and tumor necrosis factor-α, are involved in endothelial and vascular dysfunctions [25], and higher IL-6 levels are associated with an increased risk of dementia [26]. Visceral fat accumulation is associated with hyperintensity in white matter of the brain, which is a risk factor for dementia [27,28]. One study has suggested that visceral fat accumulation leads to deep white matter hyperintensity through an increase in inflammatory cytokines [28]. Excessive abdominal obesity may increase the secretion of inflammatory cytokines, which may contribute to cognitive decline.

Furthermore, adipose tissue excretes adipokines, such as leptin and adiponectin, that modulate cognitive functions [29]. Leptin functions in the homeostatic regulation of energy balance and metabolism [30] and has a neuroprotective function by regulating hippocampal synaptic plasticity and suppressing amyloid β accumulation [29]. Adiponectin has an anti-inflammatory effect and has been suggested to have a potentially neuroprotective function similar to leptin [31]. However, in conditions of obesity, despite increased leptin production, the blood–brain barrier (BBB) permeability of leptin is decreased due to the development of leptin resistance [29,32]. The production of adiponectin is also reduced under conditions of excessive abdominal obesity [29]. Therefore, the protective effects of adipokines against cognitive decline may be weakened under conditions of excessive abdominal obesity.

Brain insulin resistance might also have a potential mechanism of action. Insulin in the blood can cross the BBB into the central nervous system through an insulin receptor [33]. The density of insulin receptors is the highest in brain areas related to cognitive functions, such as the hypothalamus and hippocampus [34]. Thus, it is important to properly transport insulin into the brain; however, in obese individuals, the transport of insulin may be decreased owing to BBB dysfunction via inflammation [34]. A previous study reported that plasma insulin levels increased with increasing WC, whereas the cerebrospinal fluid/plasma insulin ratio was negatively associated with WC [35]. Therefore, reduced blood-to-brain insulin transport owing to excessive fat accumulation may cause cognitive decline.

In the present study, although abdominal fat accumulation was associated with cognitive decline, VFA in women was not associated with cognitive decline. Previous cross-sectional studies have reported that higher visceral fat levels are associated with a lower risk of cognitive impairment and MCI, suggesting that visceral fat may protect cognitive function in postmenopausal women [15,36]. One possible explanation for this phenomenon is the effect of estrogen. Estrogen, a major sex hormone for women, is involved in promoting neuroplasticity and neurogenesis in the hippocampus [37,38] and regulating Aβ production [39], suggesting the protective effect of estrogen against cognitive decline. Epidemiological studies have reported that higher serum estradiol levels are associated with a decreased risk of cognitive decline and Alzheimer’s disease (AD) development among older women [40,41]. In general, estrogen is secreted from the ovaries, and adipose tissue also plays a role as a source of estrogen because aromatase, which converts androgens into estrogens, is abundant in adipose tissues [42]. A study using brain tissue from older women suggested that peripheral adipose tissue may be a source of estrogen in postmenopausal women [43]. A study on postmenopausal women also reported that increasing visceral fat rather than subcutaneous fat could increase estrogen exposure [44]. Therefore, older women with visceral fat accumulation have higher estrogen levels, which may be protective against cognitive decline.

This study reported that higher WC, SFA, and VFA in men and higher WC and SFA in women were associated with greater cognitive decline. These results have suggested the practical/clinical implications that WC, SFA, and VFA in men and WC and SFA in women may be useful as screening to predict cognitive decline. In particular, measuring WC may be a convenient tool for predicting cognitive decline among community-dwelling older adults because WC is low-cost, simple to use, time-efficient, and easy to interpret. Moreover, the minimum value for the highest group of WC, which showed a greater decline in cognitive function compared with the lowest group, was 89.1 cm for men and 89.3 cm for women. These values closely align with the cut-off values for WC in the diagnostic criteria of metabolic syndrome (85 cm for men and 90 cm for women) in Japan [45]. In addition, the results of this study were adjusted for fat-free mass as a confounding factor, indicating the importance of maintaining low abdominal fat in later life, independent of muscle mass. However, some key factors must be considered when weight loss occurs in older adults with abdominal obesity. A randomized controlled study investigating the effect of intensive lifestyle interventions on weight loss among overweight or obese patients with type 2 diabetes, including older participants, reported that muscle mass and fat mass decreased during weight loss [46]. Consequently, adverse effects of weight loss, such as decreased bone density and increased fragility of fractures, have been reported [47]. Considering these findings, comprehensive management of body weight that considers the amount of fat-free mass is required for older adults with abdominal obesity.

This study has several methodological strengths and makes important contributions to both research and practice. First, this was a long-term longitudinal study using 10-year follow-up data. We could provide further evidence for the longitudinal relationship of visceral and subcutaneous fat with a change in cognitive function in each sex. Second, the VFA and SFA were measured using CT, which is a more objective and accurate method for assessing fat mass compared with bioelectrical impedance analysis and DXA. Third, the analysis of this study was adjusted for APOE-ε4, a genetic risk factor for AD.

The present study has some limitations. First, the findings were based on data from a certain area of Japan; therefore, a nationwide survey is required. However, our findings may be generalizable to Japanese populations aged ≥60 years without cognitive impairment because NILS-LSA participants were randomly selected from areas in Japan where most individuals lead a typical Japanese lifestyle [20]. Second, among the older adults aged ≥60 who participated in the second wave, 23.8% of older adults were excluded because they had cognitive impairment in the second wave, did not participate in any of the third to seventh waves, or had missing data for covariates. Compared with the analyzed samples, those excluded from the analysis had older age, lower cognitive function, or higher prevalence of comorbidities. Third, we could not analyze the model that included the information about the diet as a confounding factor. The previous studies have reported that a healthy diet protects against cognitive decline [48,49]. Further research investigating the influence of cognitive decline should include information about diet. Fourth, this study showed the statistically significant association that higher abdominal adiposity was associated with a greater decline in MMSE over 10 years, but the actual MMSE score change was small. The previous study investigated what a meaningful change in MMSE is among clinic patients and reported that at least 2–4 points change in MMSE indicated a reliable change over 1.5 years [50]. Therefore, we could not determine whether the observed cognitive decline was attributable to neurodegenerative diseases such as Alzheimer’s disease. Furthermore, as we could not investigate the influence on other measures of cognitive function, we cannot determine whether there were differential effects on several cognitive domains. Fifth, the prevalence of comorbidities in the participants was relatively lower compared to that in the general population of older Japanese adults. The reasons for this difference between the study participants and the general population are that comorbidities were assessed using a self-administered questionnaire [51] and that people who are concerned about their health tend to participate in research projects. Sixth, this study examined the associations with abdominal adiposity assessed at a single baseline time point; thus, the impacts of changes in abdominal adiposity on cognitive function were not considered.

## 5 Conclusions

Higher WC, SFA, and VFA in men and higher WC and SFA in women were associated with greater cognitive decline over the subsequent 10 years. However, no association was observed between visceral fat accumulation and cognitive decline in women. These findings suggest that the accumulation of abdominal adiposity is a risk factor for cognitive decline among older adults. Furthermore, the abdominal adiposity involved in cognitive decline may differ between men and women, and further studies are required to understand the mechanisms of this difference between the sexes.

## Funding statement

This work was supported by the Research Funding for Longevity Sciences (grant numbers 22-18,22-2, and22-23) from the National Center for Geriatrics and Gerontology. The funders had no role in the preparation of this manuscript.

## Conflict of interest

Kazuaki Uchida, Taiki Sugimoto, Chikako Tange, Yukiko Nishita, Hiroshi Shimokata, Naoki Saji, Yujiro Kuroda, Nanae Matsumoto, Yoshinobu Kishino, Rei Ono, Toshihiro Akisue, Rei Otsuka, and Takashi Sakurai declare no conflict of interest.

## Ethics statement

The study complied with the Declaration of Helsinki and was approved by the Ethics Committee of Human Research at the National Center for Geriatrics and Gerontology, Japan (No. 1525). All participants provided written informed consent before participating in the study.
