# Supplementary material for: Association between abdominal adiposity and cognitive decline in older adults: a 10-year community-based study
Source: J Nutr Health Aging. 2024 Feb 2;28(3):100175. doi: 10.1016/j.jnha.2024.100175 (PMC12880568; doi:10.1016/j.jnha.2024.100175)
Supplement: Supplementary file 1 [file mmc1.docx]

Supplementary Table 1. Correlation between WC, SFA, and VFA in men (n = 458)

| Variables | WC | SFA | VFA |
| --- | --- | --- | --- |
| WC | - |  |  |
| SFA | 0.860 ** | - |  |
| VFA | 0.756** | 0.605 ** | - |

* p < 0.05; ** p<0.01

Abbreviations: WC, Waist circumference; SFA, Subcutaneous fat area; VFA, Visceral fat area

Supplementary Table 2. Correlation between WC, SFA, and VFA in women (n = 415)

| Variables | WC | SFA | VFA |
| --- | --- | --- | --- |
| WC | - |  |  |
| SFA | 0.862** | - |  |
| VFA | 0.688** | 0.519** | - |

* p < 0.05; ** p<0.01

Abbreviations: WC, Waist circumference; SFA, Subcutaneous fat area; VFA, Visceral fat area

Supplementary Table 3. Comparison of the characteristics in the second wave between the older adults included the main analysis (analyzed samples) and those who met the exclusion criteria (excluded samples)

|  | Men | | |  | Women | | |
| --- | --- | --- | --- | --- | --- | --- | --- |
|  | Analyzed samples  N = 458 | Excluded  samples  N = 125 | *P value* |  | Analyzed samples  N = 415 | Excluded  samples  N = 147 | *P value* |
| Age, year | 68.76 ± 5.76 | 71.52 ± 5.54 | <0.01 |  | 68.77 ± 5.54 | 72.24 ± 5.67 | <0.01 |
| Years of education, year | 11.43 ± 2.80 | 10.62 ± 2.74 | <0.01 |  | 10.27 ± 2.22 | 9.77 ± 2.41 | 0.06 |
| missing | 0 | 0 |  |  | 0 | 1 |  |
| APOE-ε4 carriers | 87 (19.00) | 22 (17.9) | 0.78 |  | 69 (16.63) | 28 (19.31) | 0.46 |
| missing | 0 | 2 |  |  | 0 | 2 |  |
| MMSE, score | 28.07 ± 1.60 | 26.79 ± 2.53 | <0.01 |  | 28.19 ± 1.59 | 26.99 ± 2.84 | <0.01 |
| missing | 0 | 8 |  |  | 0 | 4 |  |
| BMI, kg/m^2^ | 22.84 ± 2.75 | 22.72 ± 3.22 | 0.52 |  | 22.82 ± 3.05 | 23.48 ± 3.84 | 0.17 |
| FFMI, kg/m^2^ | 17.97 ± 1.66 | 17.69 ± 1.77 | 0.09 |  | 15.79 ± 1.45 | 16.03 ± 1.74 | 0.33 |
| missing | 0 | 7 |  |  | 0 | 24 |  |
| Waist circumference, cm | 85.03 ± 8.07 | 84.90 ± 9.79 | 0.80 |  | 84.67 ± 9.47 | 86.23 ± 11.34 | 0.20 |
| Subcutaneous fat area, cm^2^ | 108.02 ± 43.89 | 111.34 ± 49.72 | 0.60 |  | 167.10 ± 64.83 | 168.71 ± 71.25 | 0.75 |
| missing | 0 | 0 |  |  | 0 | 2 |  |
| Visceral fat area, cm^2^ | 97.11 ± 54.73 | 97.20 ± 57.56 | 0.76 |  | 73.69 ± 40.02 | 85.03 ± 56.11 | 0.14 |
| missing | 0 | 0 |  |  | 0 | 1 |  |
| Height, cm | 162.66 ± 5.71 | 160.85 ± 6.08 | <0.01 |  | 149.54 ± 5.52 | 147.79 ± 6.04 | <0.01 |
| Weight, kg | 60.51 ± 8.49 | 58.93 ± 9.77 | 0.06 |  | 51.10 ± 7.72 | 51.39 ± 9.53 | 0.83 |
| Current smoker | 127 (27.73) | 45 (36.00) | 0.07 |  | 16 (3.86) | 1 (0.68) | 0.05 |
| Stroke | 33 (7.21) | 17 (13.82) | 0.02 |  | 11 (2.65) | 5 (3.47) | 0.57 |
| missing | 0 | 0 |  |  | 0 | 3 |  |
| Hypertension | 164 (35.81) | 50 (40.65) | 0.32 |  | 155 (37.35) | 70 (48.28) | 0.02 |
| missing | 0 | 2 |  |  | 0 | 2 |  |
| Dyslipidemia | 62 (13.54) | 21 (17.36) | 0.29 |  | 118 (28.43) | 45 (31.25) | 0.52 |
| missing | 0 | 4 |  |  | 0 | 3 |  |
| Diabetes mellitus | 53 (11.57) | 28 (22.58) | <0.01 |  | 27 (6.51) | 14 (9.66) | 0.21 |
| missing | 0 | 1 |  |  | 0 | 2 |  |
| Cardiovascular disease | 78 (17.03) | 27 (22.31) | 0.18 |  | 55 (13.25) | 29 (21.97) | 0.02 |
| missing | 0 | 4 |  |  | 0 | 15 |  |

*Note:* Data are presented as n (%) or mean ± standard deviation. Missing indicates the number of missing values.

Abbreviations: APOE-ε4, apolipoprotein E-ε4; MMSE, Mini-Mental State Examination; BMI, body mass index; FFMI, fat-free mass inde

Supplementary Table 4. Association between regional abdominal adiposity and change in cognitive function in men: a linear mixed model

|  | Unadjusted model | | | |  | Age-adjusted model* | | | |
| --- | --- | --- | --- | --- | --- | --- | --- | --- | --- |
|  | β | 95% CI | *P value* | P for trend |  | β | 95% CI | *P value* | P for trend |
| Waist circumference × time |  |  |  | 0.02 |  |  |  |  | 0.03 |
| Low | ref. |  |  |  |  | ref. |  |  |  |
| Middle | 0.01 | –0.12 to 0.12 | 0.99 |  |  | 0.03 | –0.08 to 0.14 | 0.62 |  |
| High | –0.15 | –0.26 to -0.03 | 0.01 |  |  | –0.12 | –0.23 to -0.01 | 0.03 |  |
| Subcutaneous fat area × time |  |  |  | 0.01 |  |  |  |  | 0.02 |
| Low | ref. |  |  |  |  | ref. |  |  |  |
| Middle | –0.05 | –0.16 to 0.07 | 0.42 |  |  | –0.04 | –0.15 to 0.08 | 0.52 |  |
| High | –0.15 | –0.27 to –0.03 | 0.01 |  |  | –0.13 | –0.24 to –0.02 | 0.03 |  |
| Visceral fat area × time |  |  |  | 0.04 |  |  |  |  | 0.04 |
| Low | ref. |  |  |  |  | ref. |  |  |  |
| Middle | 0.03 | –0.08 to 0.15 | 0.60 |  |  | 0.04 | –0.08 to 0.14 | 0.59 |  |
| High | –0.10 | –0.22 to –0.01 | 0.03 |  |  | –0.09 | –0.21 to –0.01 | 0.04 |  |

Only age was included as a confounding factor in the age-adjusted model.

Abbreviations: FFMI, fat-free mass index; APOE-ε4, apolipoprotein E-ε4

Note: In this linear mixed model, the repeated MMSE score at each wave from baseline was used as the dependent variable, and the regional abdominal adiposity index at the second wave (ref. lowest group), time, and the interaction of regional abdominal adiposity indices and time were used as the independent variables, and participant-specific intercepts and the variability of MMSE score during follow-up were considered as a random effect. Supplementary Table 4 indicates only the results of the interaction between abdominal adiposity index and time.

Supplementary Table 5. Association between regional abdominal adiposity and change in cognitive function in women: a linear mixed model

|  | Unadjusted model | | | |  | Age-adjusted model* | | | |
| --- | --- | --- | --- | --- | --- | --- | --- | --- | --- |
|  | β | 95% CI | *P value* | P for trend |  | β | 95% CI | *P value* | P for trend |
| Waist circumference × time |  |  |  | 0.03 |  |  |  |  | 0.04 |
| Low | ref. |  |  |  |  | ref. |  |  |  |
| Middle | –0.08 | –0.20 to 0.05 | 0.24 |  |  | –0.07 | –0.19 to 0.05 | 0.26 |  |
| High | –0.14 | –0.26 to –0.01 | 0.03 |  |  | –0.13 | –0.25 to –0.01 | 0.04 |  |
| Subcutaneous fat area × time |  |  |  | 0.03 |  |  |  |  | 0.03 |
| Low | ref. |  |  |  |  | ref. |  |  |  |
| Middle | –0.09 | –0.22 to 0.04 | 0.16 |  |  | –0.09 | –0.21 to 0.04 | 0.18 |  |
| High | –0.14 | –0.27 to –0.01 | 0.03 |  |  | –0.13 | –0.26 to –0.01 | 0.04 |  |
| Visceral fat area × time |  |  |  | 0.73 |  |  |  |  | 0.67 |
| Low | ref. |  |  |  |  | ref. |  |  |  |
| Middle | –0.07 | –0.20 to 0.05 | 0.26 |  |  | –0.07 | –0.19 to 0.06 | 0.28 |  |
| High | 0.02 | –0.11 to 0.15 | 0.74 |  |  | 0.03 | –0.10 to 0.15 | 0.68 |  |

*Only age was included as a confounding factor in the age-adjusted model.

Abbreviations: FFMI, fat-free mass index; APOE-ε4, apolipoprotein E-ε4

Note: In this linear mixed model, the repeated MMSE score at each wave from baseline was used as the dependent variable, and the regional abdominal adiposity index at the second wave (ref. lowest group), time, and the interaction of regional abdominal adiposity indices and time were used as the independent variables, and participant-specific intercepts and the variability of MMSE score during follow-up were considered as a random effect. Supplementary Table 5 indicates only the results of the interaction between abdominal adiposity index and time.
